# Magnetic resonance imaging guidance for the optimization of ventricular tachycardia ablation

**DOI:** 10.1093/europace/euy040

**Published:** 2018-03-23

**Authors:** Rahul K Mukherjee, John Whitaker, Steven E Williams, Reza Razavi, Mark D O’Neill

**Affiliations:** 1 School of Biomedical Engineering and Imaging Sciences, 4th Floor, North Wing, St Thomas’ Hospital, King’s College London, London, UK; 2 Department of Cardiology, Guy’s and St Thomas’ Hospital NHS Foundation Trust, London, UK

**Keywords:** Ventricular tachycardia, Cardiac magnetic resonance imaging, Substrate, Catheter ablation, Real time, Image integration, Active tracking

## Abstract

Catheter ablation has an important role in the management of patients with ventricular tachycardia (VT) but is limited by modest long-term success rates. Magnetic resonance imaging (MRI) can provide valuable anatomic and functional information as well as potentially improve identification of target sites for ablation. A major limitation of current MRI protocols is the spatial resolution required to identify the areas of tissue responsible for VT but recent developments have led to new strategies which may improve substrate assessment. Potential ways in which detailed information gained from MRI may be utilized during electrophysiology procedures include image integration or performing a procedure under real-time MRI guidance. Image integration allows pre-procedural magnetic resonance (MR) images to be registered with electroanatomical maps to help guide VT ablation and has shown promise in preliminary studies. However, multiple errors can arise during this process due to the registration technique used, changes in ventricular geometry between the time of MRI and the ablation procedure, respiratory and cardiac motion. As isthmus sites may only be a few millimetres wide, reducing these errors may be critical to improve outcomes in VT ablation. Real-time MR-guided intervention has emerged as an alternative solution to address the limitations of pre-acquired imaging to guide ablation. There is now a growing body of literature describing the feasibility, techniques, and potential applications of real-time MR-guided electrophysiology. We review whether real-time MR-guided intervention could be applied in the setting of VT ablation and the potential challenges that need to be overcome.

## Introduction

The majority of sudden cardiac deaths can be attributed to ventricular tachycardia (VT) or ventricular fibrillation.^1^ Ventricular tachycardia in the presence of structural heart disease (SHD) frequently involves re-entry around or within a region of scar. Dense scar may be caused by a previous myocardial infarction in ischaemic cardiomyopathy (ICM) whilst diffuse fibrosis may be present in non-ischaemic cardiomyopathy (NICM).^2^ Radiofrequency (RF) catheter ablation has emerged as a promising management strategy and can reduce recurrences of VT and appropriate implantable cardioverter-defibrillator (ICD) therapies in patients with both ICM^3^^,^^4^ and NICM.^5^ However, VT ablation in SHD can be associated with significant complications and modest long-term success rates.^6^ The standard approach to VT ablation uses electroanatomical mapping (EAM) and electrogram-defined surrogates of abnormal myocardium to target the regions potentially capable of sustaining re-entry. There are several limitations to this approach including the inability to interrogate the complex 3D geometry of scar, limited spatial resolution of voltage-based mapping, and failure to perform activation mapping during unstable arrhythmias without haemodynamic support.^7^

There is a clinical need to improve the efficacy of catheter ablation for VT. In this context, magnetic resonance imaging (MRI) could play a role in the optimization of VT ablation. Scar and scar borderzone (BZ) are 3D structures—MRI using late gadolinium enhancement (LGE) is the best available technique to define scar in 3D and is a powerful predictor of ventricular arrhythmia risk.^8^ Although MRI has value in patients with idiopathic VT,^9^^,^^10^ its usefulness in patients with SHD is more pronounced. In this article, we assess the role of MRI for the identification of substrate causing VT in SHD. We review the use of image integration to guide VT ablation and potential limitations of this strategy. An alternative approach to utilizing the benefits of imaging is real-time magnetic resonance (MR)-guided intervention. We assess whether real-time MRI-guided electrophysiology could play a role in VT ablation and the challenges that need to be overcome to make this a reality.

## Magnetic resonance imaging to identify the structural substrate for ventricular tachycardia

The presence of scar detected using LGE-MRI is an independent predictor of sudden cardiac death, recurrent VT, or appropriate ICD discharge in patients with a history of sustained or non-sustained VT.^11^ However, some types of scar may be more arrhythmogenic than others.

The presence of heterogeneous tissue at the BZ regions between scar and normal myocardium has been more frequently observed in patients with VT compared to patients without VT matched for age, sex, infarct location, and left ventricular ejection fraction.^12^ The challenge for MR is to enable accurate discrimination of arrhythmogenic from non-arrhythmogenic scar. Different characteristics of scar such as the location, signal intensity (SI), transmurality, shape, and complexity have all been related to the substrate for VT.

There are subtle aspects of a VT isthmus, however, that can only presently be defined using EAM including entry and exit-sites. The common channel isthmus of VT circuits may also be formed by functional rather than fixed lines of block whilst barriers forming the isthmus may not be clearly visible during sinus rhythm.^13^ It thus seems unlikely that MRI may be able to define characteristics of an isthmus using structural criteria alone but may augment the identification of arrhythmogenic substrate when EAM may be equivocal.

### Scar transmurality

The transmurality of scar has been co-localized to local electrogram characteristics, however, the relationship remains incompletely understood. A mismatch between infarct surface area measurements on MRI-reconstructed images and EAM using a <1.5 mV bipolar voltage cut-off has been reported in 33% of cases. The areas of mismatch occurred in regions such as the basal left ventricular septum or the posterior wall where achievement of catheter stability and adequate tissue contact may be more challenging during EAM.^14^

Critical sites for maintenance of VT have been confined to areas of scar with >75% transmurality and in core–BZ transition sites^15^ whilst regions of slow conduction with a stimulus-QRS time (S-QRS) >40 ms have also been associated with regions of scar with >75% transmurality.^16^ The complementary use of MRI could therefore allow improved identification of scar and scar depth that may not be well assessed using EAM alone.

### Scar localization

Endocardial bipolar voltage mapping with its limited field of view could miss epicardial substrate^17^ whilst the additive use of MRI for substrate localization could improve the sensitivity and specificity of predicting an epicardial origin for ventricular arrhythmia in NICM.^18^ Epicardial mapping can be challenging due to the presence of fat affecting the quality of recorded bipolar electrograms or may even be inaccessible due to the presence of pericardial adhesions.^19^ Magnetic resonance imaging could therefore provide detailed information on scar location non-invasively that could be used to determine access and ablation strategies.

### Signal intensity and complexity of scar

The voxel SI of scar on MR images has been used to differentiate between core areas of scar and BZ regions of scar. Using pixel SI thresholds of 60% and 40% of maximum SI to define the distribution of core scar and BZ regions, critical sites for clinical VTs have been identified on MRI when compared with EAM,^20^ albeit with significant false positive rates.^21^ In a chronic porcine infarct model, areas of grey-zone (BZ) quantified using LGE-MRI exhibited abnormal potentials more frequently than healthy tissue or dense infarct during right ventricular pacing.^22^ Recently, a new measure of scar inhomogeneity—‘entropy’ of the probability of distribution of SI within scar tissue has been found to be associated with VT burden independent of total scar and left ventricular ejection fraction.^23^ Such measures may prove to be promising discriminators between arrhythmogenic and non-arrhythmogenic scars.

### Insights from high resolution *ex vivo* imaging

**Figure 1:**
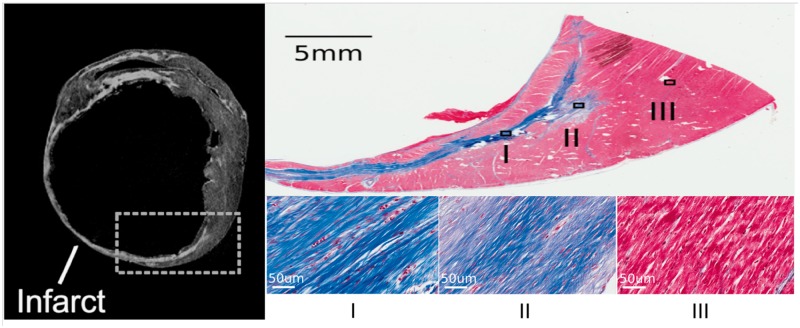
*Ex vivo* MRI and histology in a porcine chronic infarct heart using Masson’s trichrome staining. Top left panel—slice of *ex vivo* LGE image demonstrating area of hyper-enhanced infarct with thin rim of normal myocardium at the endocardial border. Top right panel—Masson’s trichrome stain of histological section demonstrating the area highlighted in the previous panel. Bottom right three panels—Three sections selected in the infarct area and control area. Blue represents collagenous scar whilst pink represents normal myocardial tissue in non-infarcted regions. Adapted with permission from Pashakhanloo *et al.*^26^ LGE, late gadolinium enhancement; MRI, magnetic resonance imaging.

Whether MRI can accurately identify the specific regions of tissue that participate in the VT circuit remains an open question. Using the Rhythmia™ high-resolution mapping system in a porcine infarct model the mean length and width of a VT isthmus were reported as 16.4 ± 7.2 mm and 7.4 ± 2.8 mm, respectively.^13^ Entrainment mapping overestimated the size of the isthmus compared with activation mapping by 32 ± 18%.^13^ Similar dimensions have also been reported in patients with conventional mapping systems (CARTO, Biosense Webster) although with a wider range of values.^24^*In vivo* MRI invariably suffers from partial volume effects due to insufficient spatial resolution whereby there is a loss of contrast between two adjacent tissue types resulting in both tissue types occupying the same voxel. This may limit the identification of scar BZ areas where the substrate for VT is likely to be located. MRI with high spatial resolution (0.39 mm^3^) has been used *ex vivo* in porcine infarct models to minimize partial volume effects and understand the fine 3D architecture of infarcts. The VT re-entry isthmus was characterized by a small volume of normal myocardium bound by scar tissue at the infarct BZ or over the infarct.^25^ Rims of surviving myocardium surrounded by scar on *ex vivo* imaging correlated on histology with normal myocardium bordered by collagenous scar (*Figure 1*).^26^ The challenge is to develop high-resolution imaging of this nature to guide VT ablation *in vivo*.

### Relationship between electroanatomical mapping defined scar and magnetic resonance imaging-derived scar: importance of bipolar voltage thresholds

Whilst some studies have reported a close correlation between areas of MR-derived scar and low bipolar voltage,^27^ others have reported a greater degree of mismatch.^14^ Although a bipolar voltage threshold of 1.5 mV is universally implemented to define ventricular scar, this was defined using single-point ablation catheters and validated in ICM.^28^ Scar areas mapped using multi-electrode mapping catheters (PentaRay) were 22% smaller using a 1.5 mV bipolar voltage threshold compared to standard 3.5 mm catheters^29^ suggesting that different catheters with various electrode sizes and inter-electrode spacing may need catheter-specific thresholds for improved scar characterization. The resolution of different mapping catheters could partially explain areas of mis-match between EAM-defined scar and MR-derived scar. Using *ex vivo* MRI as a gold standard, as bipolar spacing increased, the optimal voltage threshold to detect MRI-defined scar also increased, whilst statistically-derived 95% thresholds were not fully sensitive for the detection of non-transmural scar.^30^ These studies suggest that individualized patient-specific approaches may be necessary for scar detection as strict voltage cut-offs may not necessarily be reflective of scar biology in regions of tissue heterogeneity.^29^

### Developments in magnetic resonance techniques to improve substrate assessment

Acquisition of high-resolution imaging is required to detect smaller regions of scar and improve assessment of scar geometry in patients undergoing VT ablation.^31^ 3D LGE imaging can allow acquisitions with a higher spatial resolution compared with 2D LGE but requires prolonged scan durations. Longer scans can lead to changes in contrast agent concentration over time or reduced respirator navigator efficiency due to irregular breathing patterns.^31^ The use of image acceleration techniques such as compressed sensing has enabled faster acquisition of 3D imaging of higher spatial resolution (1.2 mm^3^) with acceptable scan duration, image quality, and comparable scar characteristics to conventional 3D LGE.^31^ Parallel imaging with a stack-of-spirals acquisition technique has also been used to enable rapid 3D LGE acquisitions in a 12 heart beat-long breath-hold.^32^ 3D free-breathing self-navigating MR sequences have recently been described to overcome the errors associated with respiratory navigator placement and irregular breathing patterns enabling high-resolution visualization of scar distribution and superior delineation of scar borders.^33^ A new technique to reconstruct a high resolution image from multiple low resolution views of the same volume (super-resolution reconstruction) has also shown a good agreement with the bipolar voltage range of scar BZ.^34^ Further developments in these techniques may allow faster imaging with higher spatial resolution and advance the ability of clinical MRI protocols to identify areas of scar critical for re-entrant VT circuits.

The development of dark-blood LGE sequences as a technical solution to sub-optimal contrast between scar and blood pool offers promise for improved substrate assessment. Dark-blood LGE sequences have been described that simultaneously reduce normal myocardium and blood pool SI whilst enhancing scar-blood contrast and preserving scar-myocardium contrast.^35^^,^^36^ Given that a large proportion of VTs appear to originate in the sub-endocardial region^37^ improved contrast between scar and blood pool may improve the detection of substrate in these areas.

### Limitations of magnetic resonance imaging for substrate assessment in patients with implantable cardioverter-defibrillators

A major limitation to more widespread use of MRI in patients undergoing VT ablation is the presence of ICDs. Although potential safety concerns have been raised when scanning patients with ICDs including tissue heating adjacent to lead electrodes, induction of arrhythmias, and alterations to device function,^38^ these risks appear to be low.^39^ In addition to safety concerns, the presence of devices can also provoke hyper-intense off-resonance artefacts mimicking scar tissue affecting the reliability of LGE imaging. The introduction of wideband LGE sequences however has improved the of artefact-free visibility of myocardial segments and enhanced the diagnostic value of the MRI exam in patients with implanted devices.^40^ A device-dependent imaging strategy with a greater use of spoiled gradient-echo sequences rather than conventional steady-state free precession imaging also appears to improve the diagnostic image quality in patients with devices.^41^^,^^42^

Despite the increasing use of MRI-compatible ICDs, not all centres have established scanning protocols in place for patients with ICDs. The in-plane spatial resolution of the wideband LGE techniques reported have also been limited to 1.5 × 1.5 mm with a typical slice thickness of 7–8 mm—this is unlikely to be sufficient for defining the fine myocardial architecture responsible for VT.^43^ It also remains unclear what effect prolonged exposure to MRI environments may have on safety in patients with devices—e.g. during real-time MR-guided interventions.

## Image integration for ventricular tachycardia ablation

**Figure 2:**
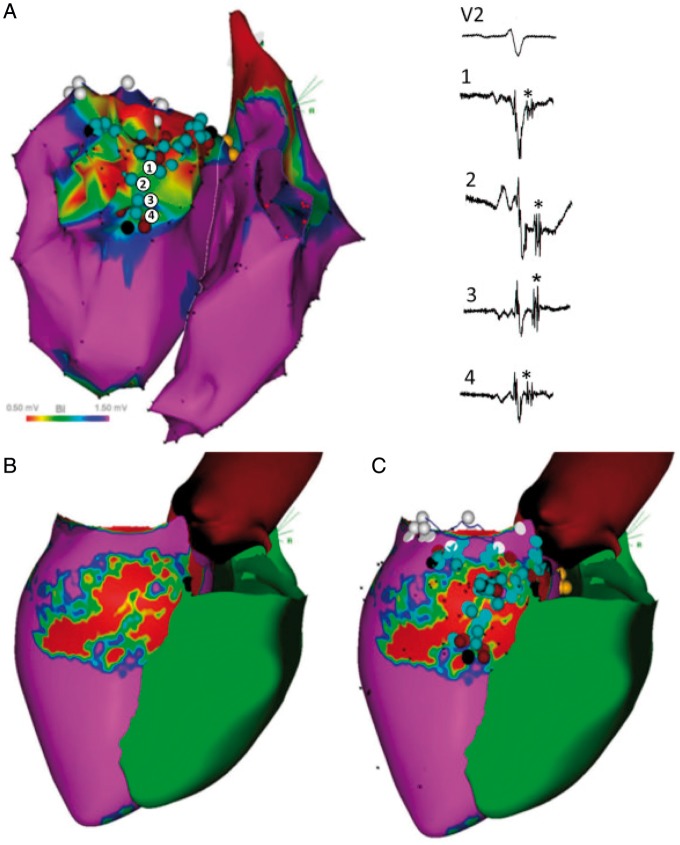
Image integration of MRI to EAM system for VT ablation. (*A*) Bipolar voltage map of a patient with ischaemic scar. Points 1–4 represent electrograms with late diastolic components. (*B*) Pixel signal intensity map derived from LGE-MRI. Areas of dense scar are shown in red, healthy tissue in pink whilst borderzone regions are shown in green/blue/yellow. (*C*) Registered LGE-MRI with EAM demonstrating corridors of conducting channels within scar tissue. Reproduced with permission from Andreu *et al.*^54^ EAM, electroanatomical mapping; LGE, late gadolinium enhancement; MRI, magnetic resonance imaging; VT, ventricular tachycardia.

Using data gained from preprocedural LGE-MRI, a number of studies have attempted to perform periprocedural registration of scar tissue with EAM through image integration (*Table 1*) and guide VT ablation (*Figure 2*). Most of these reports were derived from retrospective analyses on limited series of patients assessing the correlation between MRI-derived scar and EAM.^15^^,^^16^^,^^44–49^ Some investigators have assessed the accuracy of registration techniques^50^ or the use of automated image analysis solutions for image integration^51^ and demonstrated a high segmentation accuracy and lower inter-observer variability compared with manual image analysis. More recently, a growing number of studies have investigated the impact of image integration on procedural management,^52^^,^^53^ acute or long-term outcomes.^54^^,^^55^

**Table 1:** Image integration studies using MRI during VT ablation

References	Patient group	MRI sequence	Electroanatomic mapping	Registration method	Registration accuracy	Anatomical landmarks	Outcomes
Bogun *et al.*^82^	29 patients with NICM referred for catheter ablation of VT or PVC	2D IR turbo fast low-angle shot; spatial resolution—1.4 × 2.2 × 8.0 mm	CARTO; 3.5 mm tip open-irrigation ablation catheter	Landmark + surface; CartoMerge	4.8 ± 3.6 mm	Aorta, LV apex, mitral annulus	LGE-MRI can identify arrhythmogenic substrate in NICM and plan appropriate mapping + ablation strategy
Desjardins *et al.*^44^	14 patients with ICM	2D IR turbo fast low-angle shot; spatial resolution—1.4 × 2.2 × 8.0 mm	CARTO; 3.5 mm tip open-irrigation ablation catheter	Landmark + surface; CartoMerge	4.3 ± 3.2 mm	Aorta, LV apex, mitral annulus	Infarct depth correlated with EGM characteristics whilst critical sites for VT were confined to LGE areas
Andreu *et al.*^45^	10 patients with ICM	3D IR gradient echo sequence; spatial resolution—1.4 mm^3^	CARTO; multipolar diagnostic catheter	Landmark + surface; CartoMerge	3.4 ± 2.9 mm	LV apex, mitral annulus, aortic annulus, RV	Best match for scar core and borderzone between LGE-MRI and EAM achieved with a cut-off value of 60% of maximum pixel SI
Wijnmaalen *et al.*^46^	15 patients with ICM	3D IR turbo-field echo; slice thickness—5 mm	CARTO; 3.5 mm tip open irrigation catheter	Landmark + surface + visual alignment; CartoMerge	3.8 ± 0.6 mm	Ostium of the left main	Local bipolar and unipolar voltages decreased with increasing scar transmurality
Dickfeld *et al.*^47^	22 patients with ICDs with either ICM or NICM	2D (8 mm slice thickness) and 3D (4–6 mm slice thickness) IR sequences	CARTO; 3.5 mm open irrigation-tip catheter	Visual alignment + landmark; CartoMerge or CARTO SOUND	3.9 ± 1.8 mm	LV apex, mitral valve, RV septal insertion	LGE-MRI can be safely performed in selected patients with ICDs
Tao *et al.*^49^	26 patients with transmural scar referred for VT ablation—3 image integration methods compared to CartoMerge	3D IR turbo-field echo; slice thickness—10 mm	CARTO; 3.5 mm irrigated-tip mapping catheter	CartoMerge; Landmark, translation and translation + rotation model	4.3–6.6 mm	Ostium of left main coronary artery	No significant differences in scar correlation was observed between the three registration methods and CartoMerge
Gupta *et al.*^56^	23 patients with ICM	2D IR turbo fast low-angle shot; spatial resolution—1.4 × 2.2 × 8 mm	CARTO; 3.5 mm tip open irrigation ablation catheter	Landmark + surface; CartoMerge	3.8 ± 0.8 mm	LV apex, aorta, mitral annulus	86% of low voltage points on EAM projected onto the registered scar. All sites critical to VT circuits projected on the registered scar
Sasaki *et al.*^16^	23 patients with ICM	IR fast gradient echo; spatial resolution—1.5 × 2.0 × 8.0 mm	CARTO; 3.5 mm tip electrode irrigated ablation catheter	Retrospective registration; Landmark + surface	2.8 ± 0.7 mm	LV apex, mitral annulus, aortic annulus	Slow conduction sites with >40 ms stimulus-QRS time were associated with a >75% scar transmurality
Spears *et al.*^83^	10 patients with NICM referred for endocardial VT ablation	IR gradient echo; spatial resolution—1.3 × 1.3 × 6.0 mm	CARTO; 3.5 mm tip irrigated mapping catheter	Landmark + surface; CartoMerge	3.6 ± 2.9 mm	Aorta, His bundle, mitral valve annulus, LV apex	Bipolar voltage >1.9 mV and unipolar voltage <6.7 mV had a negative predictive value of 91% for detecting non-endocardial scar from no scar or endocardial scar
Cochet *et al.*^48^	9 patients referred for VT ablation (3 ICM, 3 NICM, 2 myocarditis, 1 idiopathic)	3D IR gradient echo; spatial resolution—1.25 × 1.25 × 2.5 mm	NavX and CARTO; PentaRay and 3.5-mm irrigated-tip catheter	Landmark + surface	NR	Coronary sinus, left atrium, mitral annulus, LV, aortic root	In ICM, areas of low voltage matched areas of LGE-MRI. In myocarditis, sub-epicardial LGE matched areas of epicardial low voltage
Piers *et al.*^19^	10 patients with NICM and VT undergoing epicardial EAM with real-time image integration	3D IR turbo field echo	CARTO; 3.5 mm irrigated tip catheter	Visual alignment + landmark; CartoMerge	3.2 ± 0.4 mm	Left main coronary artery	Bipolar voltage, unipolar voltage and electrogram duration >50 ms distinguished scar from myocardium in areas with <2.8 mm fat
Piers *et al.*^15^	44 patients referred for VT ablation (23 ICM and 21 NICM)	3D IR turbo field echo	CARTO; 3.5 mm irrigated tip catheter	Visual alignment + landmark; CartoMerge	3.8 ± 0.6 mm	Left main coronary artery	Critical isthmus sites located in close proximity to CMR-derived core–borderzone transition and in regions with >75% transmural scar
Tao *et al.*^49^	15 patients with ICM	3D IR turbo field echo	CARTO; catheter details not reported	Visual alignment + landmark; CartoMerge	4.9 ± 1.5 mm	Left main coronary artery	Cohen’s kappa coefficient between MR-defined scar and EAM-scar was 0.36 ± 0.16
Yamashita *et al.*^53^	125 patients with ICM (real-time image integration used in 38%)	NR	CARTO or NavX; Navistar or Pentaray catheter	Landmark; CartoMerge or field scaling using NavX	NR	NR	VT recurrence was observed in 36% during follow-up. Use of image integration was an independent predictor of clinical outcome
Yamashita *et al.*^52^	116 patients (67 ICM; 30 NICM; 19 ARVC) (imaging used: MDCT—91%; CMR—30%)	3D IR gradient echo; spatial resolution—1.25 × 1.25 × 2.5 mm	CARTO or NavX; Navistar or Pentaray catheter	Landmark + surface; CartoMerge or field scaling using NavX	3.9 ± 1.0 mm	NR	Image integration motivated additional mapping in 57% of patients and epicardial access in 33%
Andreu *et al.*^54^	159 patients with scar-related VT: 54 patients underwent CMR-aided ablation	3D IR gradient echo; spatial resolution—1.4 mm^3^	CARTO; 3.5 mm tip irrigated ablation catheter	Landmark + surface; CartoMerge	NR	RV, aortic root	CMR-aided ablation was an independent predictor of VT recurrence

ARVC, arrhythmogenic right ventricular cardiomyopathy; CMR, cardiac magnetic resonance; EAM, electroanatomic mapping; ICD, implantable cardioverter-defibrillator; ICM, ischaemic cardiomyopathy; IR, inversion recovery; LGE, late gadolinium enhancement; LV, left ventricle; MDCT, multiple detector computed tomography; MRI, magnetic resonance imaging; NICM, non-ischaemic cardiomyopathy; NR, not reported; PVC, premature ventricular complexes; RV, right ventricle; SI, signal intensity; VT, ventricular tachycardia.

The registration error reported within these studies have generally been in the range of 3–5 mm (*Table 1*) whilst some investigators have completed the registration process in real-time, rather than *post hoc* allowing the electrophysiologist to focus mapping efforts to regions of interest identified through imaging.^56^

In a prospective non-randomized study, Andreu *et al.*^54^ have reported on the impact of image integration on acute and long-term outcomes in VT ablation. A scar dechanneling technique was used to perform substrate-guided ablation with 34% of patients having pixel SI maps integrated into the navigation system during ablation. The use of MR guidance resulted in a lower number of RF applications and RF delivery time needed to achieve non-inducibility. There was also a lower recurrence of VT reported in the CMR-aided ablation group over a mean follow-up of 20 ± 19 months. The authors speculated that these findings may have been due to identification of arrhythmogenic substrate on CMR that was hidden on EAM and better localization of subsequent target ablation sites.^54^ It is important to note, however, that due to the lack of randomization in this study, the control group had more dilated left ventricles with longer VT cycle lengths compared to patients who underwent CMR-guided ablation. In addition, 18% of patients who underwent CMR did not have scar data post-processed for image integration due to either poor image quality or improper acquisition. A retrospective analysis in patients with dilated cardiomyopathy has also shown that the use of preprocedural MRI was associated with improved procedural success and improved survival free of a composite endpoint of VT recurrence, heart transplantation, or death over a median 7.6 months of follow-up.^55^ This report compared an imaging group with a historical control group where preprocedural imaging was not available. The findings from these studies appear promising but prospective randomized evidence is needed to assess if image integration can truly improve the efficiency and efficacy of VT ablation.

### Limitations of image integration studies

To date, all studies that have used image integration to guide procedures have relied on MRI data acquired days or weeks prior to ablation. Changes in the volume, orientation, and rhythm of the heart between the time of the initial MRI and ablation procedure may lead to inaccuracies in registering scar data from MR to EAM shells. Small discrepancies in landmark identification on imaging and EAM can further perpetuate registration errors. Translational changes due to patient movement, cardiac or respiratory motion can also lead to discrepancies between imaging and EAM.^57^ Potential registration errors of even a few millimetres, as described in the image integration studies, could lead to misidentification of critical sites with potential consequences on the efficacy of ablation.

The use of intra-procedural imaging with real-time MRI to guide ablation may potentially overcome some of these limitations and appears attractive. Magnetic resonance imaging can provide detailed information on the cardiac chamber of interest as well as surrounding structures. Respiratory and cardiac motion is also more accurately assessed by MRI than any other imaging modality—compensating for these factors during RF ablation could have potential implications for the safety and efficacy of ablation.^57^

## Real-time magnetic resonance-guided electrophysiology and ablation

Over the last decade, a number of centres have developed MR-compatible ablation systems, reliable methods for visualizing the catheter tip and application of a workflow to perform electrophysiology procedures in high magnetic field environments.^58^ Diagnostic studies have been performed in animal models to characterize intra-cardiac electrograms within ventricular scar under real-time MR-guidance^22^ whilst ablation lesions have been assessed in real-time using dedicated MR techniques.^59^ In order to develop these technologies and establish procedural workflows all studies performed in patients to date have focused on atrial flutter and ablation of the cavo-tricuspid isthmus.^60^^,^^61^ However, the most logical application of real-time MRI-guided electrophysiology would be in the setting of VT where the benefits of substrate imaging are greater. Improved assessment of ventricular substrate with accurate real-time registration of imaging to EAM data may overcome some of the limitations of current image integration approaches. Further potential benefits of real-time MR-guidance could include improved procedural guidance without exposure to radiation and improved lesion visualization and titration of therapy according to MR-defined end-points.

### Intra-procedural guidance

**Figure 3:**
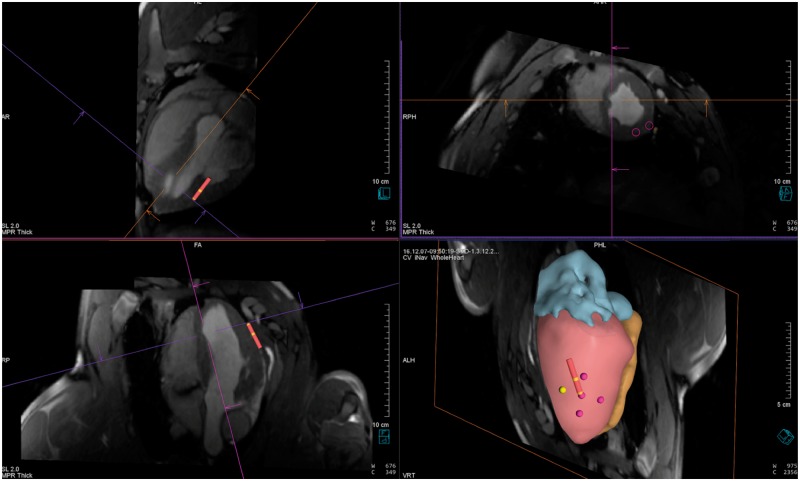
MRI for intra-procedural guidance in a porcine model during real-time intervention. Top panels and bottom left panel—three orthogonal slices through the left ventricle. The red catheter icon represents the actively tracked catheter during epicardial ablation. Bottom right panel—3D reconstructed shell of cardiac chambers (red, left ventricle; orange, right ventricle; blue, left atrium) to guide navigation based on 3D whole heart MRI acquired at the time of procedure. The points represent regions where an ablation lesion has been delivered. MRI, magnetic resonance imaging.

A potential benefit of real-time MRI guidance over conventional EAM is the ability to visualize soft tissue structures and access detailed information on the chamber of interest and surrounding structures to guide catheters to desired locations within the heart. Both passive tracking (recognition of local susceptibility artefacts induced by markers near catheter tip—e.g. dark spots) and active tracking (monitoring the active signal of a RF coil placed near the catheter tip to determine position) have been used for accurate localization and visualization of catheters during real-time MR-guided interventions (*Figure 3*).^62^^,^^63^

Dukkipati *et al.*^64^ first described the use of active catheter tracking to navigate catheters into the left ventricle in swine, record intra-cardiac electrograms and acquire 3D voltage maps. Further studies have described the use of active tracking to perform trans-septal puncture and left atrial access in an animal model.^63^^,^^65^^,^^66^ Both passive tracking^58^ and active tracking^60^^,^^61^ have been used to guide atrial flutter ablation in patients. There are as yet no studies describing the use of active tracking in patients undergoing VT ablation and although significant logistical challenges exist, this is a potential avenue of future work.

### Ablation lesion assessment

**Figure 4:**
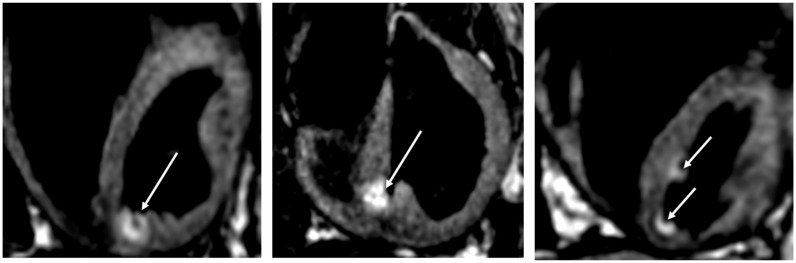
Acute assessment of ablation lesions using non-contrast T1-weighted MRI in a porcine model. A hyper-intense region is seen in the myocardium corresponding to the location of ablation lesion delivery (white arrows). The advantage of non-contrast techniques to assess ablation lesions would be to allow repeated assessments during an index procedure and potentially perform additional ‘top-up’ ablation if required. MRI, magnetic resonance imaging.

Magnetic resonance imaging can be used to visualize lesions in real-time, acutely (*Figure 4*) and assess chronic injury. Lardo *et al.*^67^ first described the use of MRI to characterize the spatial and temporal extent of ablation lesions in mongrel dogs. Subsequent work has described distinct phases of signal enhancement over time of contrast-enhanced lesion imaging^68^ and the optimal timing of late-enhancement imaging to accurately predict chronic lesion volume.^69^ During real-time MR-guided interventions, contrast can only be given once however, necessitating the development of non-contrast sequences for lesion visualization. Both T1-weighted and T2-weighted non-contrast techniques have been described to visualize ablation lesions with high-signal areas in lesion cores and surrounding low intensity rims corresponding on histopathology to central tissue necrosis and a transition zone, respectively.^70^

The ability to clearly visualize ablation injury using MRI could offer a potential new procedural endpoint to judge if sufficient ablation has been performed. In a porcine model of chronic MI, the ability to induce VT was associated with a substantially larger zone of heterogeneous tissue. Animals then underwent ablation with at least one lesion delivered in the heterogeneous zone. A repeat MRI and EP study performed 1 week after ablation showed that animals that were still inducible had remaining areas of heterogeneous tissue due to incomplete ablation. In two animals without remaining heterogeneous tissue on MRI, VT was not inducible.^71^ This is in keeping with clinical experience where an extensive substrate-based approach targeting all abnormal electrograms has been reported to be superior, in terms of VT recurrence, to limited ablation strategies targeting only clinical and stable VTs.^72^ Assessment of remaining heterogeneous tissue on MRI following ablation may prove to be a useful imaging endpoint during real-time MRI-guided ablation.

Visualization of ablation lesions hours or weeks after a procedure does not allow modification of the ablation strategy at the index procedure. Just as the electrophysiology community seeks improved lesion assessment techniques, there is growing interest in MR-based techniques to visualize lesions in real-time informing RF delivery and potentially improving the efficacy of ablation. MR-thermometry using the proton resonance frequency shift (PRFS) has emerged as a useful technique in this regard. Protons are surrounded by electrons which act to disturb the local magnetic field during MR scanning.^57^ Following ablation there is an increase in temperature, breaking of inter-molecular hydrogen bonds and an increased density of free electrons. The change in PRFS using phase mapping has been used to quantify tissue temperature changes during ablation and demonstrated in mongrel dogs.^73^ More recently, in an ovine model, endocardial ablation could be clearly monitored in real-time using MR-thermometry with thermal lesion dimensions highly correlated to measurements at gross macroscopy.^74^

Online monitoring of cardiac temperature during real-time MR-guided ablation appears promising, but there are several challenges to overcome including adequate compensation for artefacts due to respiratory or cardiac motion, assessing accuracy of the technique during irregular heart rhythms and investigating the ability of the technique to predict chronic transmural injury. This tool however, represents an interesting step in exploiting acute physiological changes during ablation using MR to potentially titrate RF energy delivery.^57^ Further validation work is warranted to see if the technique is feasible in patients during real-time MR-guided ablation.

### Other interventional considerations and potential challenges for magnetic resonance-guided ventricular tachycardia ablation

Assessment of substrate and delivery of RF energy are only two components of a catheter ablation procedure for VT. Epicardial access or emergency pericardiocentesis may be required during VT ablation—a previous report in a porcine model described the feasibility of real-time MRI-guided subxiphoid pericardial access and pericardiocentesis using passive needle devices^75^ but no clinical studies to demonstrate safety have been performed to date.

Transcatheter needle chemoablation to target intra-mural substrate has generated interest to improve the efficacy of ablation in selected patient cohorts. In this regard, Rogers *et al.*^76^ have delivered chemoablation with ethanol or acetic acid under real-time MRI guidance in swine with immediate visualization of lesions and subsequent EAM confirmation of conductive isthmus disruption which appears promising.

The advent of novel emerging technologies such as stereotactic body radiation therapy (SBRT) to deliver ‘radioablation’ and create electrically inert tissue further increases the importance of accurate, non-invasive characterization of substrate. In a recent case series, SBRT was used to deliver catheter-free radioablation to patients with refractory VT with a marked reduction in the burden of VT in all patients.^77^ The development, refinement and validation of real-time MRI techniques could be a critical component in developing such novel therapies.

There is currently a lack of commercially available MR-compatible external defibrillator systems. The need to defibrillate urgently during VT ablation and the potential time required to evacuate a patient from a MR scanner bore and perform external defibrillation is a major safety hurdle that needs to be overcome in order to facilitate the uptake of higher-risk MR-guided ablation procedures. In this context, a recent report has described a prototype MR-conditional defibrillation system to successfully defibrillate swine inside and outside a scanner bore.^78^ Electrical cardioversion during a procedure will inevitably result in patient movement and subsequent changes in the position of the heart relative to reconstructed anatomical shells. As is the case with EAM systems, repeated real-time MR confirmation scans may be necessary to update anatomical shells and maintain accurate registration.^58^ New acquisitions with 3D reconstructions would prolong what is already likely to be a long procedure.

Contemporary electrophysiology procedures in complex arrhythmias are increasingly guided by 3D EAM systems. Current generation MR-compatible catheters cannot achieve the same resolution as multipolar mapping catheters used with high-resolution 3D EAM systems.^79^ A potential workflow to performing real-time MRI-guided intervention could therefore be to perform a conventional electrophysiological assessment followed by moving the patient into the MRI scanner for intra-procedural assessment of imaging substrate, guidance with active tracking, ablation lesion delivery and lesion assessment under real-time MR guidance. Development of such a workflow will require rapid, automated segmentation and registration tools and could allow the benefits of conventional EAM assessment to be combined with real-time imaging but can realistically only be performed in hybrid X-ray and MRI catheter laboratories.

Radiofrequency-induced tissue heating is an important safety concern particularly when scanning patients with ICDs and it remains unclear what the safety implications may be when performing long procedures under MRI guidance in these patients. In patients with devices that are not MRI-compatible, alternative imaging modalities such as cardiac computed tomography to assess VT substrate (wall thinning and scar) may be more appropriate.^80^ In order to enable fast image analysis during real-time MR-guided interventions, rapid segmentation, registration and visualization platforms are required, which are currently under active development.^81^

Other challenges that need to be addressed include safe anaesthesia and monitoring of patients and ensuring good communication between the electrophysiologist in the MR environment and other healthcare professionals involved in the procedure. Although some of these issues have been navigated in the setting of atrial flutter ablation, performing VT ablation under real-time MR-guidance adds a new dimension of complexity. There is also potentially a steep learning curve for an electrophysiologist in handling and manoeuvring new MR-compatible catheters as well as interpreting MR images for which they may have received little training. Although there is much interest within the electrophysiology community in real-time MR-guided interventions, there are relatively few centres with the resources, expertise and technologies to perform such interventions.^57^

## Future perspectives

Despite the substantial progress that has been made on the use of MRI to guide VT ablation, there is insufficient evidence at present to suggest that the use of imaging can add value to clinical outcomes. Although observational, non-randomized studies suggest that image integration may impact on procedural outcomes, well-designed prospective randomized studies are needed to assess the true impact of image integration as well as evaluate potential mechanisms of any benefit. Further studies evaluating new MRI techniques to distinguish between arrhythmogenic and non-arrhythmogenic scar at high spatial resolution may have downstream effects on improving patient selection for catheter ablation and increased standardization of ablation strategies based on imaging evaluation. Increased standardization in the management of these patients may help reduce workflow discrepancies across centres and potentially impact on the efficacy of ablation.

Real-time MRI-guided electrophysiology is a field still in its relative infancy. Further technical development and validation studies are required to improve substrate localization in real-time, corroborate the accuracy of procedural guidance with active tracking and assess the ability of real-time techniques to predict durable ablation lesions. Pre-clinical and clinical studies will be required to standardize workflows for real-time MRI-guided interventions and ultimately compared with conventional VT ablation in order to demonstrate added value.

## Conclusion

There has been significant progress in the use of MRI to facilitate substrate assessment, integrate data into clinical EAM systems, guide procedures and assess ablation lesions. There is however a lack of prospective randomized evidence to assess if image integration using MR improves the efficacy or efficiency of ablation. There are also a number of technical limitations to using an image integration approach to guide VT ablation. The possibility of real-time MR-guided ablation is intriguing but to date has only been studied in pre-clinical models and patients undergoing atrial flutter ablation. Catheter ablation for VT is likely to reap the greatest benefits from developments in real-time MR-guided electrophysiology but significant challenges need to be addressed prior to clinical studies in patients.

## Funding

This work was supported by the National Institute for Health Research (NIHR) Biomedical Research Centre at Guy’s and St Thomas’ NHS Foundation Trust and King’s College London as well as a Wellcome Trust Health Innovation Challenge Fund grant (HICF-R10-698). Whitaker is a recipient of a Fellowship from the Medical Research Council (MR/N001877/1). The views expressed in this manuscript are those of the authors and not those of the NIHR, MRC, or NHS.

**Conflict of interest:** none declared.
